# Paliperidone as an Alternative Treatment for Behavioral Symptoms in Childhood Autism: Case Report of an Eight-Year-Old Patient

**DOI:** 10.7759/cureus.90262

**Published:** 2025-08-16

**Authors:** Gizem Keles, Augustine Rajakumar, Crystal M Wilson

**Affiliations:** 1 Behavioral Health, Lake Erie College of Osteopathic Medicine, Bradenton, USA; 2 Academic Research Services, BayCare Health System, Tampa, USA; 3 BayCare Behavioral Health, BayCare Health System, Tampa, USA

**Keywords:** aripiprazole, attention deficit hyperactivity disorder (adhd), autism spectrum disorder (asd), child and adolescent psychiatry, disruptive mood dysregulation disorder (dmdd), intermittent explosive disorder, paliperidone, risperidone

## Abstract

This case report discusses an eight-year-old boy diagnosed with autism spectrum disorder (ASD), attention-deficit/hyperactivity disorder (ADHD), and disruptive mood dysregulation disorder (DMDD), who required two separate psychiatric hospitalizations within a four-month period due to severe aggression and self-harming behaviors. The patient was initially admitted involuntarily under the Florida Mental Health Act of 1971 after exhibiting violent behavior toward family members and significant self-harm. During his first hospitalization, which lasted 25 days, he demonstrated a limited response to multiple combination interventions, including stimulants, α-agonists, and antipsychotics such as risperidone and olanzapine. Paliperidone, an atypical antipsychotic typically used in adolescents for schizophrenia, was titrated up to 3 mg/day, resulting in significant improvement in irritability and aggression with no immediate adverse effects. Four months after discharge, the patient was readmitted for similar behaviors, including aggression toward teachers and renewed self-harm. Despite adherence to his prescribed medications during the interim period, his symptoms returned abruptly, one week prior to readmission. During this second hospitalization, lasting 16 days, the dose of paliperidone was increased to 3 mg twice daily, based on the Florida Best Practice Psychotherapeutic Medication Guidelines for Children and Adolescents, leading to rapid resolution of aggressive episodes. The patient tolerated the dosage adjustment without significant side effects, though weight gain remained a concern. This case highlights paliperidone’s potential utility in managing severe behavioral dysregulation in pediatric patients with ASD when first-line therapies fail. It also underscores the importance of careful monitoring for metabolic side effects in this vulnerable population. Further research is needed to establish age-specific dosing guidelines and long-term safety profiles for children under 12 years of age.

## Introduction

Paliperidone, an atypical antipsychotic approved for schizophrenia in adults and adolescents aged 12-17 [1] and for schizoaffective disorder in adults [1], has seen limited but growing use in pediatric populations for severe behavioral comorbidities associated with autism spectrum disorder (ASD). While risperidone and aripiprazole are first-line treatments for irritability in ASD [2,3], paliperidone’s active metabolite status (9-hydroxyrisperidone) and once-daily dosing offer potential advantages in adherence [1]. However, its safety profile in children, particularly under 12 years, remains less established. Common side effects include weight gain, dizziness, and sedation [1,4], while serious risks include metabolic abnormalities, hyperprolactinemia, and movement disorders such as tardive dyskinesia [1,4-7]. Neuroleptic malignant syndrome (NMS) and QT prolongation are rare but critical considerations [4-7]. Retrospective studies in adolescents demonstrate tolerability, though younger children may face heightened vulnerability to metabolic and endocrine effects [4-7].

This case report examines the use of paliperidone in an eight-year-old boy with ASD, attention-deficit/hyperactivity disorder (ADHD), and disruptive mood dysregulation disorder (DMDD). Children with ASD and comorbid psychiatric conditions often present with complex behavioral challenges, including aggression and self-injury, which can be difficult to manage [8,9]. While psychiatric medications are typically studied for isolated conditions using symptom-specific rating scales, this case highlights the real-world complexity of treating overlapping symptoms across multiple diagnoses. The patient was followed across two psychiatric hospitalizations within a four-month period due to escalating behavioral dysregulation.

## Case presentation

Patient history

The patient is an eight-year-old boy with a complex psychiatric history and developmental delays. He was born at 40 weeks’ gestation via vaginal delivery without complications or NICU admission. However, both speech and motor milestones were delayed. He has no known medical conditions, surgical history, or allergies.

Psychiatrically, he has been receiving ongoing outpatient psychiatric care and therapy for over three years. He previously completed a nine-week residential psychiatric hospitalization at age seven in a different state, and per his primary caregiver, he has been hospitalized a total of 15 times for psychiatric reasons in various states. He has no history of suicide attempts.

The family psychiatric history is significant for anxiety on the maternal side and schizophrenia on the paternal side. His older brother has ASD and ADHD, and both parents also have ADHD. There is no family history of substance use disorders or suicide.

Of note, his parents divorced a year prior. The patient lives with his mother and maternal grandmother, while his older brother is away at college. He enjoys interacting with peers but only when activities are on his terms. He has no concerns about gender identity and prefers activities such as video games, Legos, and coloring. There is no history of abuse. Academically, the patient is a third-grade student with failing grades. He has received two in-school suspensions but has not needed to repeat a grade year.

Initial admission

At the time of admission, the patient carried diagnoses of ASD, ADHD, and DMDD. He was admitted to a psychiatric recovery center involuntarily through the Florida Mental Health Act of 1971 (Baker Act) due to violent behavior toward family members as well as risk of self-harm. Prior to hospitalization, the patient exhibited near-daily episodes of physical aggression and self-injurious behaviors, which significantly impaired his ability to attend school, disrupted family dynamics, and created ongoing safety concerns at home. His home medications at the time included atomoxetine 18 mg once daily for ADHD [10], clonidine 0.1 mg twice daily for ADHD [10], and olanzapine 2.5 mg daily for mood dysregulation symptoms [10]. Aside from medications, the patient had previously participated in applied behavior analysis (ABA), engaged in behavioral therapy, was actively followed by an outpatient psychiatrist, and had a prior stay at a residential treatment center (RTC) for long-term behavioral stabilization.

Although a Baker Act mandates a 72-hour hold, the patient remained hospitalized for 25 days due to a number of factors, including difficulty stabilizing the patient, finding a new RTC that would take the patient, and ongoing safety concerns with individuals within the household. During his stay, the patient received daily behavioral therapy in both individual and group formats, and participated in regular meetings with social services and therapeutic staff to support behavioral stabilization and discharge planning, in addition to pharmacologic management.

During the patient’s nearly month-long stay, the patient exhibited frequent tantrums that escalated to aggressive behavior toward staff and self-harm (e.g., slamming his head on the walls, kicking doors with the intent of breaking his foot), necessitating the use of restraints on several occasions. This pattern of behavior was consistent with the severe irritability and aggression reported by caregivers prior to admission, as reflected in the initial Aberrant Behavior Checklist irritability subsection (ABC-I) score of 34/45 and the Clinical Global Impression-Severity (CGI-S) rating of 5 (“markedly ill”). 

The Aberrant Behavior Checklist-Irritability subscale (ABC-I) is a validated caregiver-rated tool used to assess irritability, aggression, and self-injury in youth with ASD and ADHD [11]. It includes 15 items scored 0-3, with a maximum of 45 [11]. The patient’s ABC-I score of 34/45 indicated severe behavioral dysregulation. The Clinical Global Impression-Severity (CGI-S) is a clinician-rated measure of overall illness severity, scored from 1 (normal) to 7 (most extremely ill); the patient’s score of 5 reflects marked impairment [12]. Both scales are commonly used in pediatric neuropsychiatric research and clinical care to track treatment response [11,12].

Baseline growth chart values for height, weight, and body mass index (BMI) were collected (Table 1). Labs, including general metabolic markers, lipid panel, and endocrine values, were collected for monitoring purposes (Table 2). 

**Table 1:** Patient’s weight, height, and BMI between both admissions based on CDC growth charts for age and sex. For weight and height [13]: <5th percentile = below average; 5th–84th percentile = normal range; 85th–94th percentile = above average; ≥95th percentile = significantly above average For BMI [13]: <5th percentile = underweight; 5th–84th percentile = healthy weight; 85th–94th percentile = overweight; ≥95th percentile = obese; ≥120% of the 95th percentile or ≥45 kg/m² = severe obesity BMI: body mass index

Admission age	Measurement	Value	Percentile	Interpretation
First admission				
Eight years of age	Weight	28.6 kg	76th	Normal (5th-84th)
	Height	124.46 cm	30th	Normal (5th-84th)
	BMI	18.5 kg/m^2^	89th	Overweight (85th-94th)
Second admission				
Eight years, five months of age	Weight	32.5 kg	91st	Above average (85th-94th)
	Height	132.08 cm	79th	Normal (5th-84th)
	BMI	18.6 kg/m^2^	88th	Overweight (85th-94th)

**Table 2:** Patient’s lab values from initial admission followed by his second admission. Values provided that are relevant to prescribing psychiatric medication for a baseline. Endocrine and lipid panels were not repeated for the second admission. BUN: blood urea nitrogen; ALT: alanine aminotransferase; AST: aspartate transaminase; TSH: thyroid-stimulating hormone; HDL: high-density lipoprotein; LDL: low-density lipoprotein

Metabolic monitoring	First admission	Second admission	Reference range
Glucose	83 mg/dL (fasting)	135 mg/dL (not specified if fasting)	60-100 mg/dL
BUN	13 mg/dL	14 mg/dL	5-18 mg/dL
Creatinine	0.675 mg/dL	0.654 mg/dL	0.37-0.62 mg/dL
BUN/creatinine ratio	19.25	21.40	14-34 mg/dL
Alkaline phosphatase	286 IU/L	282 IU/L	150-409 IU/L
ALT	11 IU/L	16 IU/L	0-29 IU/L
AST	32 U/L	30 U/L	0-60 IU/L
Prolactin	7.70 ng/mL	-	5-20 ng/mL
TSH	1.66 uIU/mL	-	0.600–4.840 uIU/mL
Triglycerides	229 mg/mL	-	0-74 mg/dL
HDL	50 mg/mL	-	>39 mg/dL
LDL	73 mg/mL	-	0-109 mg/dL

The patient had a history of trialing several medications for his behavioral challenges, although he was ultimately unresponsive to methylphenidate, dexmethylphenidate, aripiprazole, guanfacine, risperidone, olanzapine, oxcarbazepine, and hydroxyzine. Details regarding dosing, duration, and clinical rationale for initiation or discontinuation were limited, as this information was obtained via collateral report from the patient’s primary caregiver and not through shared medical records from current or previous outpatient psychiatrists. As such, a comprehensive medication summary detailing dose rationale, treatment duration, and reasons for discontinuation is not available.

During this admission, medication changes were made based on the Florida Best Practice Psychotherapeutic Medication Guidelines for Children and Adolescents (Table 3) [10].

**Table 3:** Timeline and summary of medication trials, titrations, and clinical response during the 25-day inpatient admission. Medications were adjusted sequentially based on symptom severity and tolerability. ADHD: attention-deficit/hyperactivity disorder; BID: twice daily; CGI-S: Clinical Global Impression-Severity; ER: extended release; ETO: emergency treatment order

Admission day(s)	Medication(s)	Dose and frequency	Response	Action / reason for change
Days 1–7	Atomoxetine + Clonidine + Olanzapine	Atomoxetine: 18 → 36 mg/day. Clonidine: 0.1 mg BID	Decreased hyperactivity, increased outbursts	Atomoxetine reduced back to 18 mg/day due to worsening behavior
Days 8–14	Olanzapine + Atomoxetine + Clonidine	Olanzapine: 2.5 mg once → BID. Atomoxetine: 18 mg/day Clonidine: continued	CGI-S remained at 5; no improvement; required emergency doses (ETO)	Olanzapine discontinued (ineffective)
Days 15–20	Paliperidone + Atomoxetine + Clonidine	Paliperidone: 1.5 mg/day (ER). Atomoxetine: 18 mg/day. Clonidine: continued	Gradual improvement of CGI-S from 5 to 4.	Titrated paliperidone to 3 mg/day
Days 21–25	Paliperidone + Atomoxetine + Clonidine	Paliperidone: 3 mg/day (ER). Atomoxetine: 18 mg/day. Clonidine: continued	CGI-S improved from 5 to 3; outbursts ↓ to 1/week	Maintained at discharge

Atomoxetine was increased from 18 mg to 36 mg daily to address ADHD symptoms and low frustration tolerance. This titration was made in accordance with weight-based prescribing guidelines, not exceeding the recommended maximum dose of 1.4 mg/kg/day [10]. However, this adjustment led to decreased hyperactivity but increased frequency of outbursts over seven days. Atomoxetine was subsequently reduced back to 18 mg once daily.

Olanzapine was increased from 2.5 mg once daily to twice daily but failed to adequately control his aggression over seven days. This dosing remained well within recommended guidelines, which suggest a maximum of 15 mg/day for children aged six to <13 years [10]. Despite these changes, the patient still had outbursts requiring seclusions and restraints with emergency treatment orders (ETO) of olanzapine 5 mg. 

The decision to initiate paliperidone treatment was influenced by the patient’s history of previous positive response to the medication, as suggested by caregivers. This prior experience with paliperidone 3 mg/day is noteworthy as it had been discontinued due to significant weight gain, 20 lbs. (9.07 kg), over three months - a common side effect of atypical antipsychotics in pediatric populations [4]. Despite this history, the potential benefits were deemed to outweigh the risks given the severity of the patient’s symptoms and the lack of response to other medications. 

Paliperidone was initiated at 1.5 mg/day using the extended release (ER) formulation for five days and titrated to extended release 3 mg/day, with the goal of behavioral stability, targeting his aggressive outbursts. This titration followed guidelines recommending an initial dose of 1.5 mg/day and a maximum of 6 mg/day for children aged six to <13 years [10]. Aggressive episodes decreased from daily occurrences to one weekly incident. His CGI-S score improved from 5 (“markedly ill”) to 3 (“mildly ill”) within the next five days. 

The patient was discharged after achieving behavioral stability with no outbursts for over five consecutive days. His discharge medications included paliperidone ER 3 mg once daily, clonidine 0.1 mg twice daily, and atomoxetine 18 mg once daily. At discharge, he was referred for outpatient psychiatric follow-up and behavioral therapy, including applied behavior analysis (ABA). A follow-up appointment with his outpatient psychiatrist was scheduled for one week post-discharge, with continued coordination planned for community-based behavioral services and school reintegration support.

Readmission

Four months after discharge, the patient was readmitted under similar circumstances due to escalating aggression toward a teacher and renewed self-harm behaviors (e.g., punching walls). Caregivers reported that he had been stable for several months post-discharge with no outbursts or significant behavioral concerns. There were no adverse effects noted from the paliperidone or other medications the patient was on. According to his mother, the patient had been handling frustrations well, asking for help when needed, and had been asking to go to school earlier to work on assignments. In addition, there were no concerns for nonadherence to medications, nor were there psychiatric medication changes. He was still taking atomoxetine 18 mg once daily, clonidine 0.1 mg twice daily, and paliperidone ER 3 mg once daily. The patient was actively going to outpatient psychiatry appointments, though had not been approved for a residential treatment facility due to insurance issues. 

However, one week prior to readmission, his aggression returned. Both the patient and his caregivers noted that “it felt like the medication stopped working.” The mother denied any identifiable triggers and described the behavior as unpredictable over the preceding week. At the same time frame, the patient started having different food preferences and difficulty with sleep. Notable contextual changes included a recent one-week visit with his father (who also observed no concerning behavior), and the mother had a recent surgery. 

At the time of readmission, the patient had an active bilateral acute otitis externa infection and was completing a 7-day course of fluoroquinolone ear drops, which had been initiated prior to admission. The medication was discontinued within the first few days of hospitalization following completion of the prescribed course. There were no other medication changes noted. His behaviors included tantrums, breaking objects, physical assaults on teachers/caregivers, urinating on people/objects, and self-harming actions. 

Upon Readmission

The patient's ABC-I score was 36/45, slightly higher than during his initial admission. His CGI-S rating was again 5 (“markedly ill”). Initial physical examination on readmission showed a height of 132.08 cm, a weight of 32.5 kg, and a BMI of 18.6 kg/m2 (Table 1). Lipid profile and endocrine markers were not repeated during this admission, but other values repeated are glucose, BUN, creatinine, and general liver function values (Table 2).

Given the recurrence of severe aggression despite adherence to medications, paliperidone was increased from 3 mg once daily to 3 mg twice daily on admission day 2 [10]. Emergency treatment orders (ETOs) were required twice during this admission: olanzapine 5 mg IM and diphenhydramine 50 mg IM were administered for acute agitation.

The patient’s other psychiatric medications (atomoxetine 18 mg once daily and clonidine 0.1 mg twice daily) were not altered or discontinued during this admission. However, the fluoroquinoline ear drops were discontinued following resolution of the infection, which was within the first few days of admission. 

Outcomes

The patient tolerated the increased paliperidone dose without any reported adverse effects, including insomnia, appetite changes, suicidal ideation, or hallucinations. Atomoxetine 18 mg once daily and clonidine 0.1 mg twice daily were continued throughout the admission alongside paliperidone. Non-pharmacologic interventions included daily behavioral therapy sessions, both individual and group-based, as well as regular meetings with social services to support discharge planning, including family sessions. No changes were made to his therapeutic regimen during the admission aside from the paliperidone adjustment. Aggressive episodes resolved entirely within five days of the dose increase, and his CGI-S score improved from 5 (“markedly ill”) to 3 (“mildly ill”) by discharge.

The patient was discharged after a 16-day hospital stay, with follow-up care coordinated through social services and continuation of outpatient psychiatric support, including a psychiatry follow-up scheduled for one week post-discharge. At discharge, he was prescribed paliperidone ER 6 mg/day, atomoxetine 18 mg/day, and clonidine 0.1 mg twice daily. His weight at discharge had increased from 32.5 kg at admission to 32.6 kg. The patient and his caregiver were also provided with education on medication adherence, warning signs of relapse, and strategies to support emotional regulation at home. A safety plan was reviewed, and the patient was referred for continued behavioral therapy in the outpatient setting. The social services team was actively looking for a residential treatment program for the patient, but he had been denied from most due to age and severity. 

## Discussion

Paliperidone, an atypical antipsychotic, has shown promise in managing severe behavioral issues in pediatric and adolescent patients with ASD [14-16], as demonstrated in this case report of an eight-year-old boy with complex comorbidities. While paliperidone is not FDA-approved for use in children under 12, its potential efficacy in treating irritability and aggression in ASD warrants further investigation. This case highlights the challenges in managing severe behavioral symptoms in pediatric ASD patients and the need for careful consideration of benefits and risks when using atypical antipsychotics in this population. Although this patient was re-admitted to the same facility within four months, it is important to note that it was an uneventful four months with no outbursts and a functional patient. 

Currently, the primary FDA-approved pharmacological treatments for irritability in pediatric patients with ASD are risperidone [3] and aripiprazole [2]. Risperidone has demonstrated efficacy in reducing irritability, aggression, and self-injurious behavior in children with ASD [3,5,7,16]. In a randomized, double-blind trial, risperidone showed a 56.9% reduction in irritability scores compared to 14.1% for placebo [17]. Similarly, aripiprazole has proven effective in managing irritability associated with ASD in children and adolescents [2,5,18]. Clinical studies have shown significant improvements in irritability, hyperactivity, and stereotypies among those taking aripiprazole compared to placebo [2,5,18].

However, these medications are not without adverse effects. Weight gain is a significant concern with both risperidone and aripiprazole [2-4,6,17]. Although aripiprazole is often favored for its reportedly more favorable metabolic profile, findings from a study of antipsychotic-naïve youth revealed weight and fat mass increases that were comparable to those seen with risperidone [19]. The study evaluated changes at four, eight, and 12 weeks, demonstrating progressive and clinically significant gains. By 12 weeks, youth receiving risperidone gained an average of 5.3 kg, while those on aripiprazole gained 4.4 kg. Notably, this included fat mass increases of 2.45 kg (risperidone) and 2.43 kg (aripiprazole). Other common adverse effects include sedation, extrapyramidal symptoms, and metabolic disturbances [6,20]. More severe, albeit rare, adverse events include neuroleptic malignant syndrome and tardive dyskinesia [6,21].

Paliperidone, being the active metabolite of risperidone, shares a similar adverse effect profile [16]. Common side effects in pediatric patients include sedation, weight gain, hyperprolactinemia, and metabolic abnormalities such as dyslipidemia and increased risk for type 2 diabetes [1,16]. Hyperprolactinemia may result in galactorrhea, gynecomastia, and disruptions in growth and sexual maturation [1]. 

More serious adverse effects include extrapyramidal symptoms (EPS) such as dystonia, hyperkinesia (including akathisia), tremor, Parkinsonism, and dyskinesia [1,16,22]. These occur more frequently in children and adolescents than in adults, and their incidence increases with higher doses [1,22]. In adolescent schizophrenia trials, up to 40% of patients receiving 12 mg/day experienced EPS-related adverse events, compared to 0-25% at lower doses [1]. Dystonia, which may present as muscle spasms, oculogyric crisis, or dysphagia, is particularly more common in younger children and in males [1].

Additional concerns include cardiovascular effects, such as persistent tachycardia and dystonic reactions, which have been reported in children under 12 years of age [18]. 

However, its once-daily dosing may offer advantages in terms of adherence [7,14]. A recent retrospective chart review of paliperidone palmitate (the long-acting injectable form) in youth with ASD and/or intellectual disability showed promising results in terms of safety and efficacy [23]. The study reported statistically significant improvements in Clinical Global Impressions scales and reduced hospital visits, with no significant change in BMI over an average treatment duration of 21.1 months [23].

This case report has several limitations. First, specific details regarding the patient’s previous outpatient medication trials, including exact dosages, durations, and documented responses, were unavailable. Medication history was obtained via caregiver report, as records from previous providers were not accessible. Second, because this was an inpatient psychiatric setting, the primary objective was short-term behavioral stabilization. As such, long-term medication management strategies and outcomes could not be assessed.

## Conclusions

Paliperidone is a reasonable second-line option for managing severe behavioral dysregulation in children and adolescents with ASD, particularly when first-line treatments are ineffective. While evidence for its safety and efficacy is stronger in adolescents, emerging data and limited case reports suggest potential benefit in children under 12 years of age. However, younger pediatric patients may be less likely to report adverse effects, increasing their risk of undetected complications. This underscores the importance of thorough physical examinations and appropriate laboratory monitoring to support early detection of side effects. Further randomized controlled trials are needed to establish age-specific dosing and safety guidelines for this population. This case demonstrates the potential utility of paliperidone in children under 12, while reinforcing the need for careful monitoring and individualized treatment planning.

## References

[1] (2025). Paliperidone - paliperidone tablet, extended release [package insert] U.S. Food and Drug Administration website. https://nctr-crs.fda.gov/fdalabel/services/spl/set-ids/528a962a-4924-4c48-93ce-6ab1f019e52c/spl-doc?hl=paliperidone.

[2] (2025). Aripiprazole - aripiprazole tablet [package insert] U.S. Food and Drug Administration website. https://nctr-crs.fda.gov/fdalabel/services/spl/set-ids/99747897-22fe-4bee-9942-6349c0c8f6b0/spl-doc?hl=aripiprazole.

[3] (2025). Risperidone - risperidone, film coated [package insert] U.S. Food and Drug Administration website. https://nctr-crs.fda.gov/fdalabel/services/spl/set-ids/07d43bf6-a695-453a-a5c7-9581effe585c/spl-doc?hl=risperidone#S14.4.

[4] Dayabandara M, Hanwella R, Ratnatunga S, Seneviratne S, Suraweera C, de Silva VA (2017). Antipsychotic-associated weight gain: management strategies and impact on treatment adherence. Neuropsychiatr Dis Treat.

[5] Loy JH, Merry SN, Hetrick SE, Stasiak K (2017). Atypical antipsychotics for disruptive behaviour disorders in children and youths. Cochrane Database Syst Rev.

[6] Krill RA, Kumra S (2014). Metabolic consequences of second-generation antipsychotics in youth: appropriate monitoring and clinical management. Adolesc Health Med Ther.

[7] Kowalski JL, Wink LK, Blankenship K, Habenicht CD, Erickson CA, Stigler KA, McDougle CJ (2011). Paliperidone palmitate in a child with autistic disorder. J Child Adolesc Psychopharmacol.

[8] Fitzpatrick SE, Srivorakiat L, Wink LK, Pedapati EV, Erickson CA (2016). Aggression in autism spectrum disorder: presentation and treatment options. Neuropsychiatr Dis Treat.

[9] Steenfeldt-Kristensen C, Jones CA, Richards C (2020). The prevalence of self-injurious behaviour in autism: a meta-analytic study. J Autism Dev Disord.

[10] (2023). Florida best practice psychotherapeutic medication guidelines for children and adolescents. 2022-2023. Tallahassee, FL: Florida Department of Children and Families.

[11] Stoddard J, Zik J, Mazefsky CA, DeChant B, Gabriels R (2020). The internal structure of the aberrant behavior checklist irritability subscale: implications for studies of irritability in treatment-seeking youth with autism spectrum disorders. Behav Ther.

[12] Busner J, Targum SD (2007). The clinical global impressions scale: applying a research tool in clinical practice. Psychiatry (Edgmont).

[13] (2023). Centers for Disease Control and Prevention. Clinical Growth Charts. CDC. https://www.cdc.gov/growthcharts/clinical_charts.htm.

[14] Stigler KA, Erickson CA, Mullett JE, Posey DJ, McDougle CJ (2010). Paliperidone for irritability in autistic disorder. J Child Adolesc Psychopharmacol.

[15] Stigler KA, Mullett JE, Erickson CA, Posey DJ, McDougle CJ (2012). Paliperidone for irritability in adolescents and young adults with autistic disorder. Psychopharmacology (Berl).

[16] Naguy A, Adel T, Almazeedi I (2019). Paliperidone use in child psychiatry: evidence or diffidence?. Pharmacology.

[17] McCracken JT, McGough J, Shah B (2002). Risperidone in children with autism and serious behavioral problems. N Engl J Med.

[18] Coustals N, Ménard ML, Cohen D (2021). Aripiprazole in children and adolescents. J Child Adolesc Psychopharmacol.

[19] Correll CU, Manu P, Olshanskiy V, Napolitano B, Kane JM, Malhotra AK (2009). Cardiometabolic risk of second-generation antipsychotic medications during first-time use in children and adolescents. JAMA.

[20] Oshikoya KA, Carroll R, Aka I, Roden DM, Van Driest SL (2019). Adverse events associated with risperidone use in pediatric patients: a retrospective biobank study. Drugs Real World Outcomes.

[21] Iffland M, Livingstone N, Jorgensen M, Hazell P, Gillies D (2023). Pharmacological intervention for irritability, aggression, and self-injury in autism spectrum disorder (ASD). Cochrane Database Syst Rev.

[22] Singh J, Robb A, Vijapurkar U, Nuamah I, Hough D (2011). A randomized, double-blind study of paliperidone extended-release in treatment of acute schizophrenia in adolescents. Biol Psychiatry.

[23] Simpson S, Dominick KC, Erickson CA, Lamy M (2024). Safety and efficacy of paliperidone palmitate in pediatric patients with autism and intellectual disability. J Autism Dev Disord.

